# A new beacon for optics: Light Office in Türkiye and its opening forum

**DOI:** 10.1038/s41377-026-02423-1

**Published:** 2026-07-10

**Authors:** Chenzi Guo, Yuhong Bai, Sedat Nizamoğlu

**Affiliations:** 1 Light Publishing Group, Changchun Institute of Optics, Fine Mechanics and Physcis, Chinese Academy of Sciences, Changchun, China; 2 Department of Electrical and Electronics Engineering, Koç University, Istanbul, Türkiye

**Keywords:** Atom optics, Imaging and sensing

Optics and photonics is no longer confined to localized or individual laboratories. It is reshaping global communications, advanced manufacturing, healthcare diagnostics, and energy systems worldwide. For *Light: Science & Applications*, building international connections and engaging with local scientific communities have long been our central priorities.

To strengthen connections with the optics and photonics community in Türkiye, *Light: Science & Applications*, its sister journals, as well as Koç University jointly launched the Light Office in Türkiye on 22^nd^ June, accompanied and celebrated by an opening symposium.

The symposium marked a new chapter for expanding the collaborative horizons between Türkiye optics and Light family journals. National and international researchers, faculty, and students were brought under one roof in a format designed to foster deep scientific exchange, strategic vision, and meaningful partnerships.

The symposium was chaired by Prof. Sedat Nizamoğlu - Chair of the Department of Electrical & Electronics Engineering at Koç University and Head of Light Office in Türkiye, Prof. Yuhong Bai – founder of Light: Science & Applications and Light Publishing Group, Dr. Chenzi Guo – Deputy Head of Light Publishing Group and Director of eLight joined the event to represent the journals. The full-day program featured an outstanding lineup of plenary addresses by esteemed global scholars—including Prof. Aydogan Ozcan - Chancellor’s Professor at University of California, Los Angeles and Co-Editor-in-Chief of eLight, Prof. Hakan Ürey – Vice president and Chair Professor at Koç University, Prof. Andrey Rogach – Chair Professor from City University of Hong Kong, Prof. Ekmel Özbay, Chair Professor at Bilkent University. Invited talks were delivered by pioneer researchers from across the region, including Prof. Arif Engin Çetin from Izmir Biomedicine and Genome Center, Prof. Onur Tokel from Bilkent University, Prof. Onur Ferhanoğlu from Istanbul Technical University, Prof. Emir Salih Mağden from Koç University, Prof. Serhat Tozburun from Izmir Biomedicine and Genome Center, Prof. Uğur Teğin from Koç University, Prof. Serkan Ateş from Sabancı University, and Prof. Alper Kiraz from Koç University.

Dozens of posters were presented, showcasing the breadth and vitality of Turkish research in optics and photonics. The day concluded with an interactive open Q&A forum with Light family journals, during which the thoughtful questions and insightful reflections from participants and students were particularly impressive (Figs. 1–11).

**Fig. 1:**
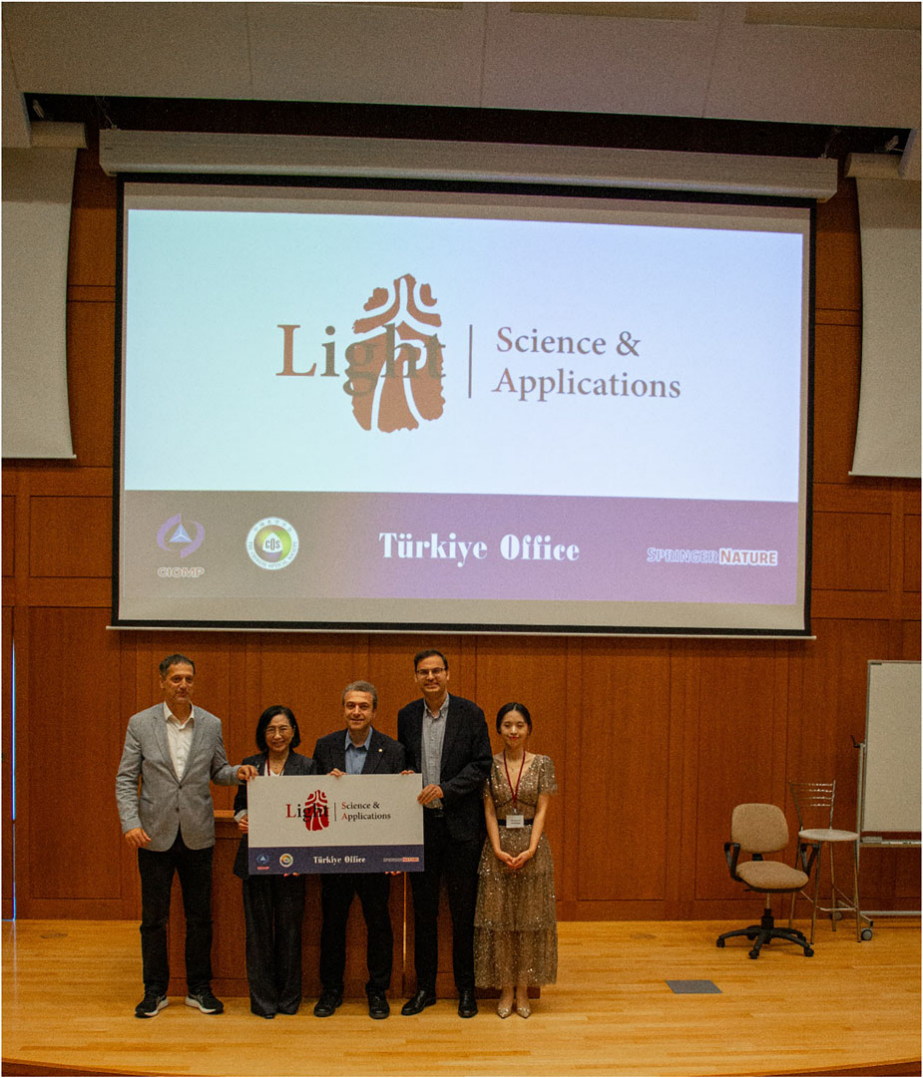
Announcement of Light Office in Türkiye. Left to right: Prof. Hakan Ürey, Prof. Yuhong Bai, Prof. Aydogan Ozcan, Prof. Sedat Nizamoğlu, Dr. Chenzi Guo

**Fig. 2:**
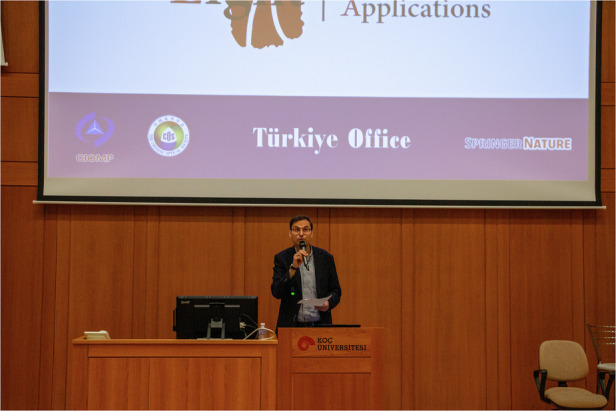
Symposium Chair: Prof. Sedat Nizamoğlu

**Fig. 3:**
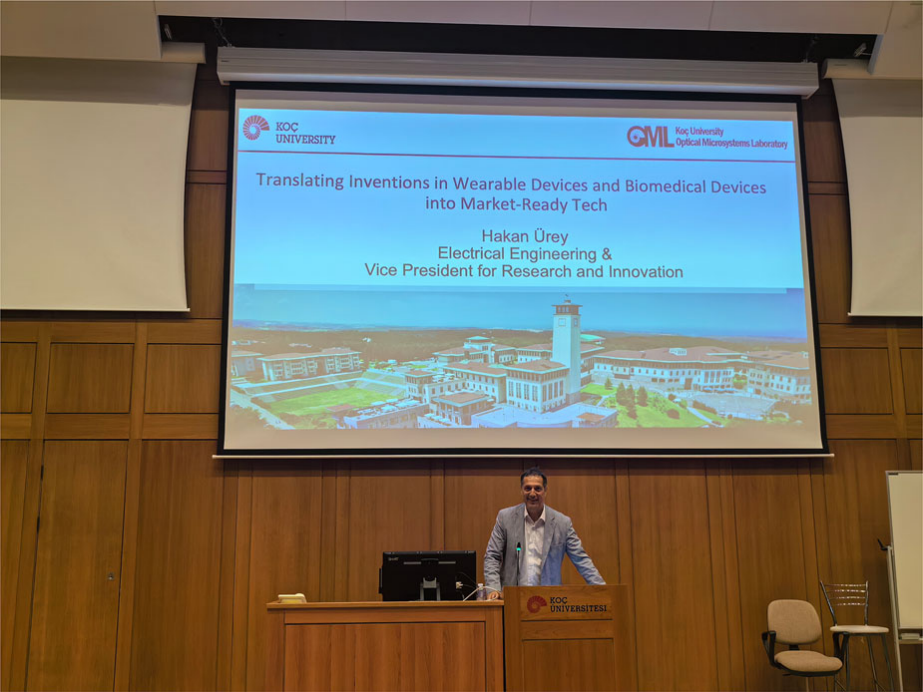
Plenary Speaker: Prof. Hakan Ürey

**Fig. 4:**
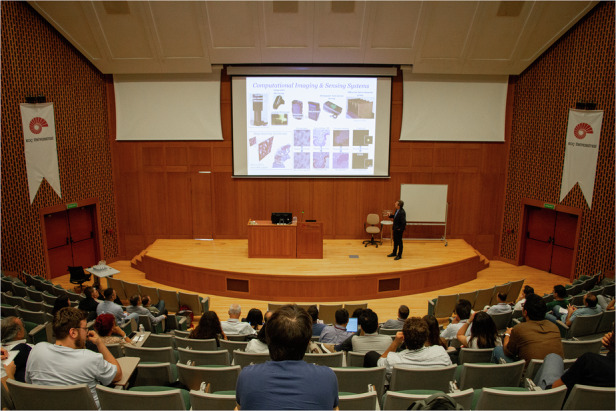
Plenary Speaker: Prof. Aydogan Ozcan

**Fig. 5:**
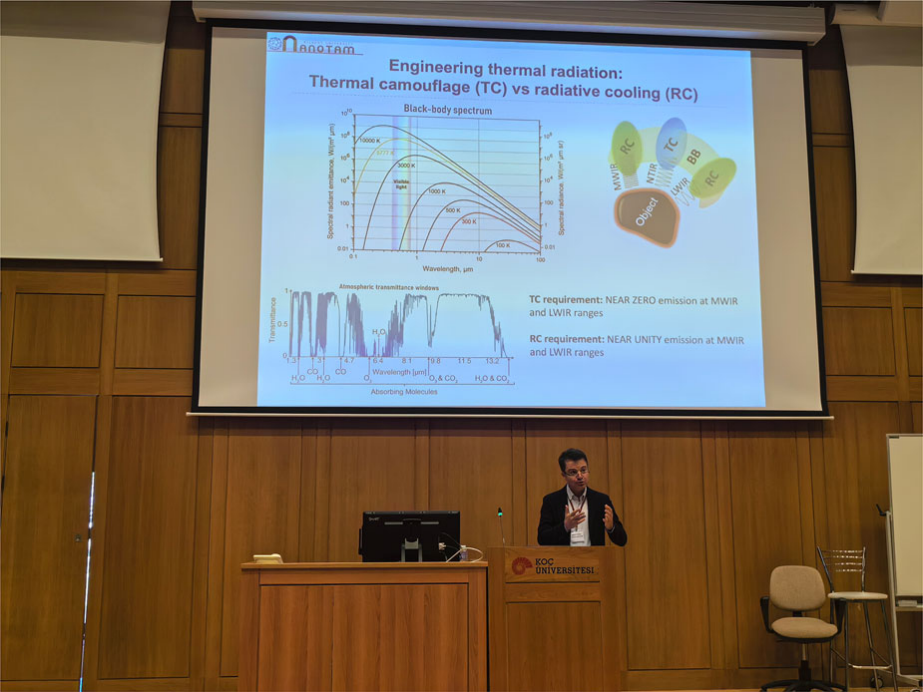
Plenary Speaker: Ekmel Özbay

**Fig. 6:**
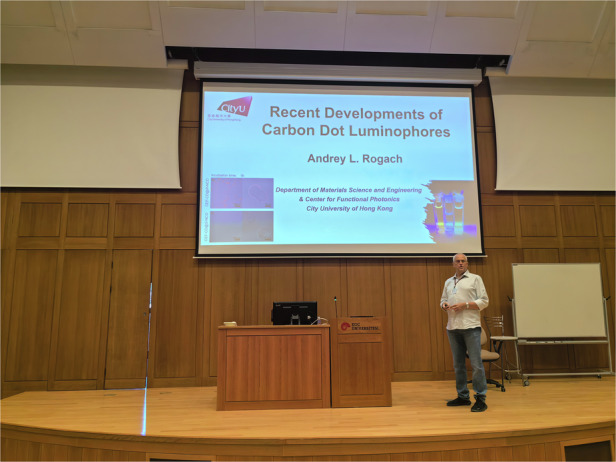
Plenary Speaker: Andrey Rogach

**Fig. 7:**
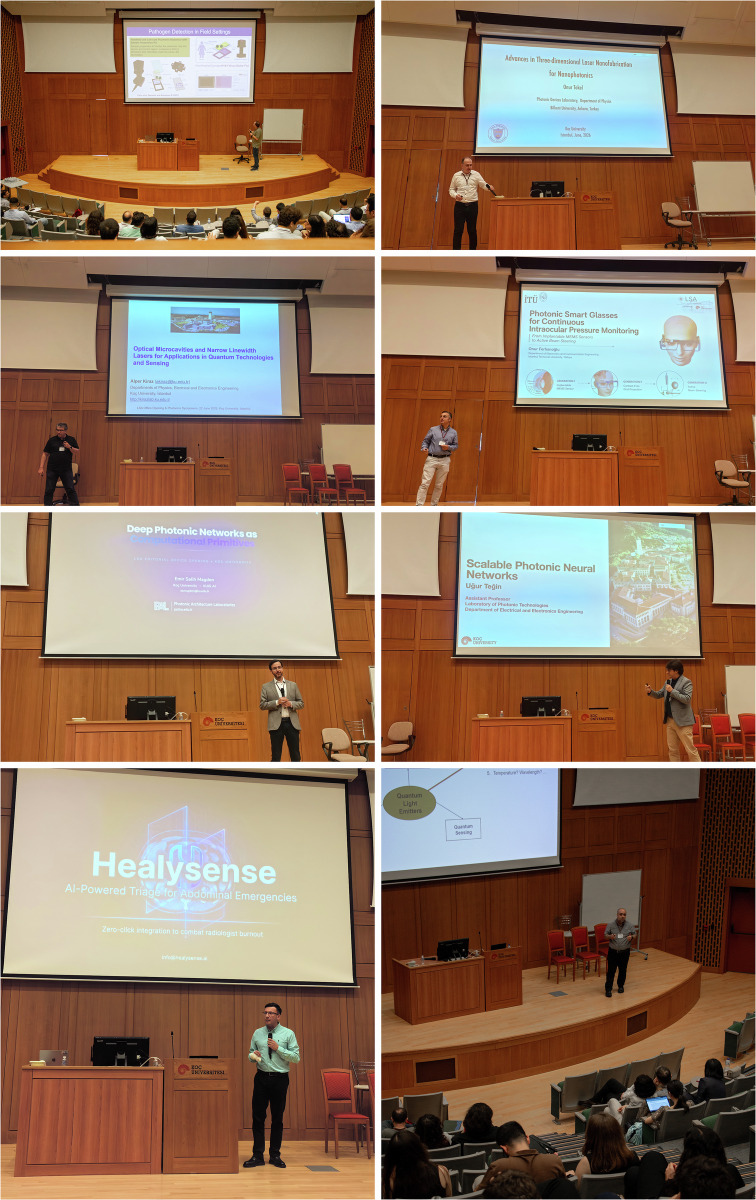
Invited session features

**Fig. 8:**
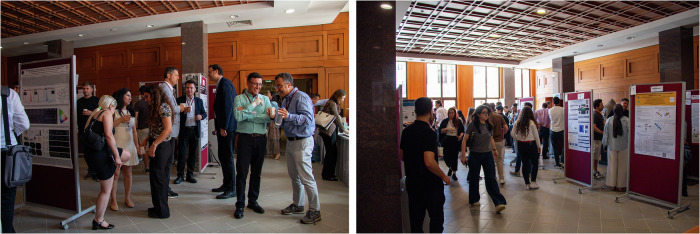
Poster Session

**Fig. 9:**
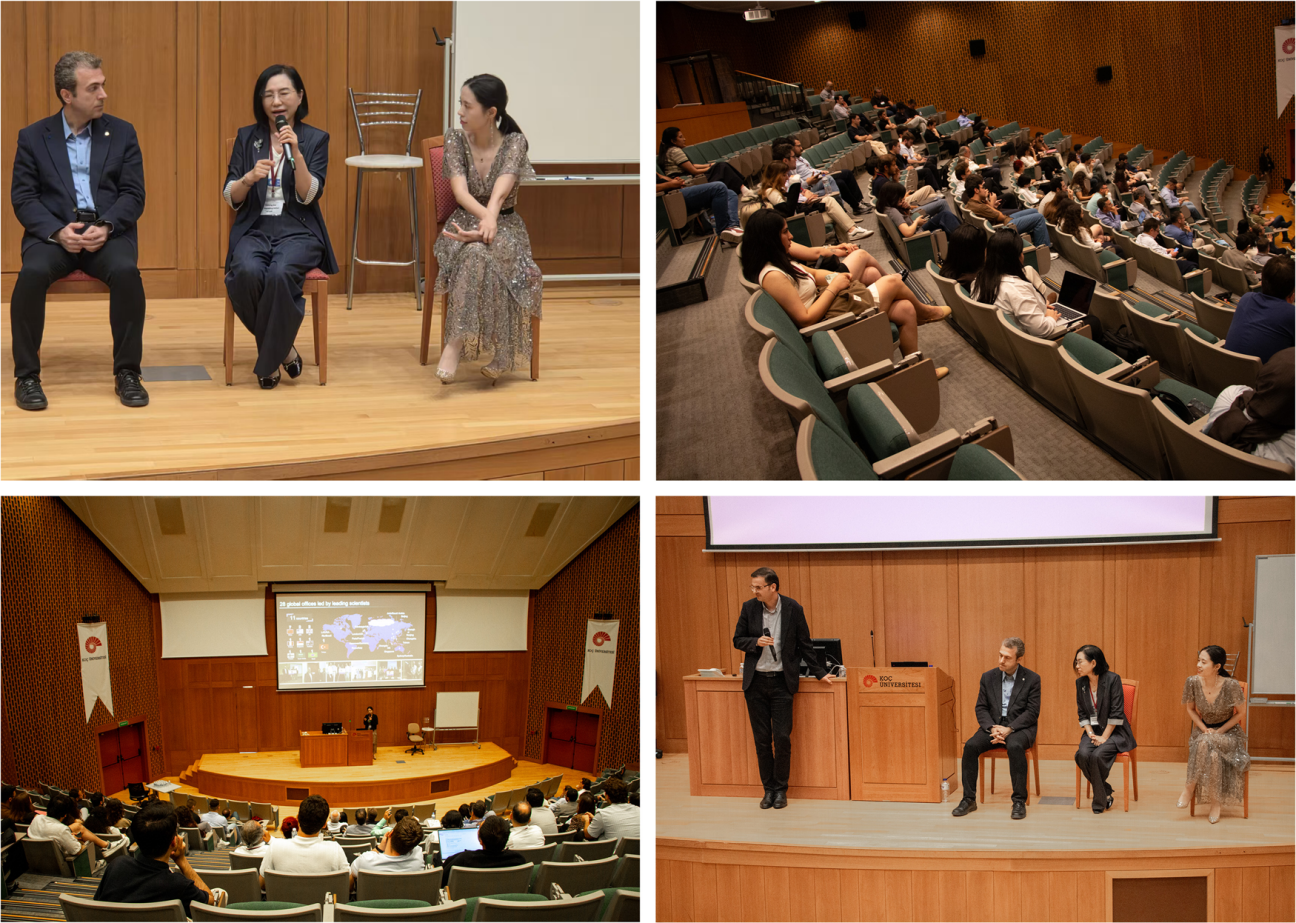
Q & A session with Light family journals & Dr. Chenzi Guo presenting the Light family journals

**Fig. 10:**
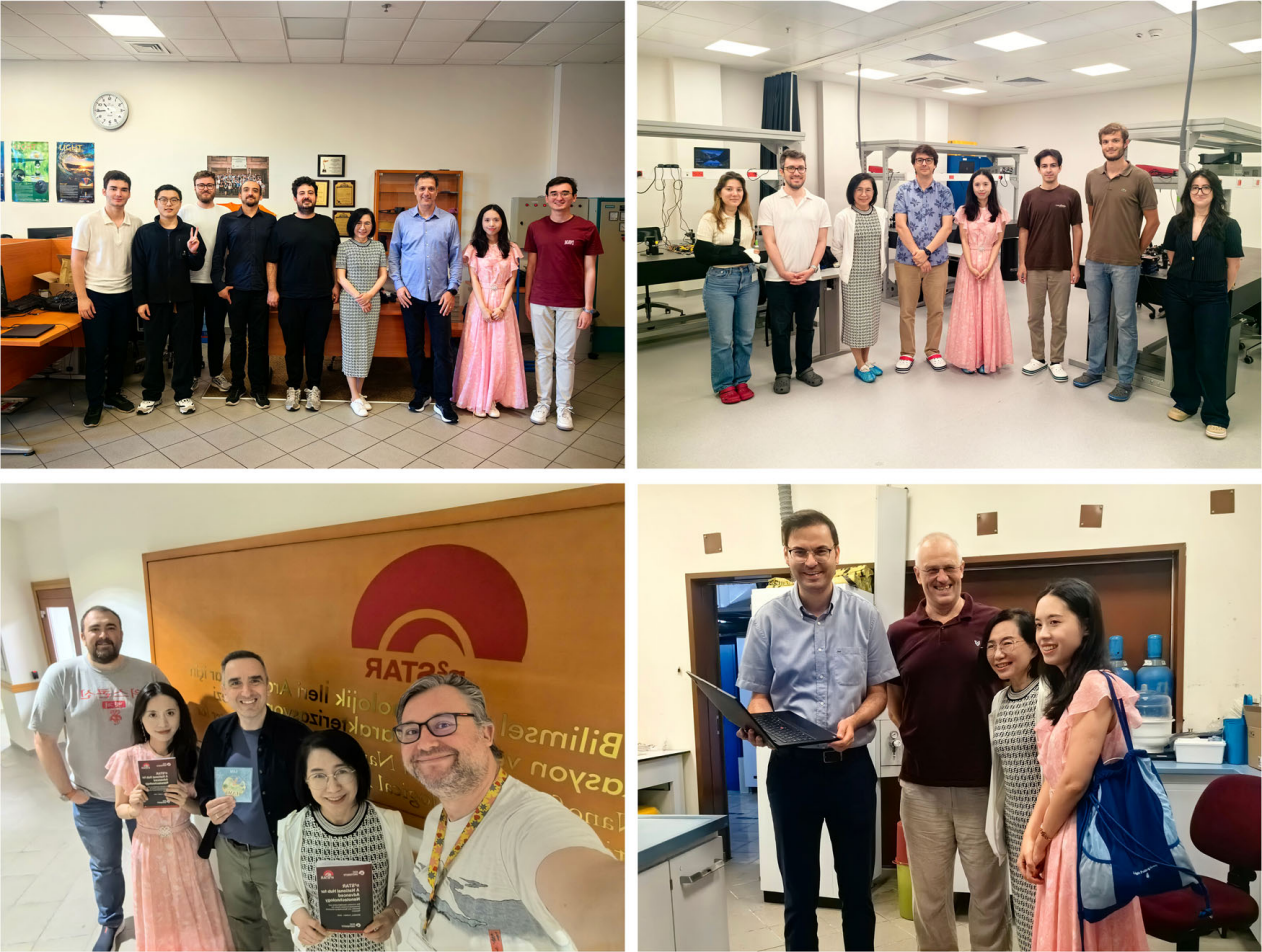
Lab visits

**Fig. 11:**
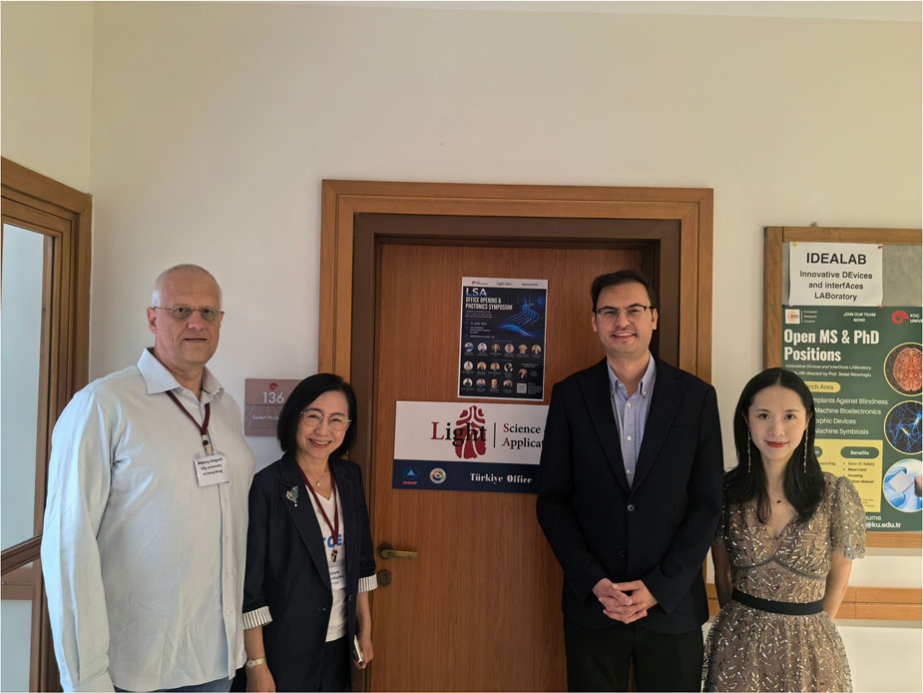
Light Office in Türkiye

As part of the event, Light family journals also visited several state-of-the-art laboratories at Koç University. The visits highlighted advanced research achievements and equipment in areas including, but not limited to, biophotonics, optical displays, optical computing, integrated photonics, and nanofabrication techniques.

As a field that sits at the vanguard of modern physics and engineering, optics and photonics demands continuous and interdisciplinary dialog, and the Light office in Türkiye is poised to become a new beacon in global optics community, resonating with the country’s flourishing optics and photonics landscape.

